# Two‐way inhibition of PAX5 transcriptional activity by PAX5::CBFA2T3

**DOI:** 10.1002/2211-5463.70087

**Published:** 2025-07-11

**Authors:** Reina Ueno, Aki Terasaki, Yuiko Imai, Yuri Kimura, Yuna Kojima, Misa Irie, Koya Odaira, Mina Noura, Shuichi Okamoto, Takahiko Yasuda, Shinobu Tsuzuki, Hitoshi Kiyoi, Fumihiko Hayakawa

**Affiliations:** ^1^ Division of Cellular and Genetic Sciences, Department of Integrated Health Sciences Nagoya University Graduate School of Medicine Japan; ^2^ Clinical Research Center National Hospital Organization Nagoya Medical Center Japan; ^3^ Department of Biochemistry Aichi Medical University School of Medicine Nagakute Japan; ^4^ Department of Hematology and Oncology Nagoya University Graduate School of Medicine Japan

**Keywords:** acute lymphoblastic leukemia, fusion gene, histone deacetylase, PAX5

## Abstract

PAX5 promotes B‐cell differentiation by transcriptional activation of B‐lineage‐specific genes. Chromosomal rearrangements in PAX5 account for 2–3% of B‐ALL cases, and most lead to the expression of in‐frame fusion transcripts. These fusions can encode chimeric proteins composed of the N‐terminal portion of PAX5 and the C‐terminal region of a variety of heterogeneous fusion partners. We analyzed the function of PAX5::CBFA2T3 (PAX5‐C), a fusion protein found in B‐cell acute lymphoblastic leukemia. PAX5‐C strongly repressed PAX5 transcriptional activity in luciferase assays. In co‐immunoprecipitation assays, PAX5‐C bound to PAX5 and HDAC1/3. However, neither HDAC knockdown nor treatment with a HDAC inhibitor showed any effect on the repression of PAX5 transactivity by PAX5‐C. In addition, PAX5‐C with DNA binding‐defective mutations (PAX5 M‐C) could still repress PAX5 transactivity; however, the repression of PAX5 transactivity by PAX5 M‐C was abolished by inhibition or knockdown of HDAC. These findings indicate that PAX5‐C exhibits two mechanisms of repression: a DNA binding‐dependent and a HDAC‐dependent mechanism, with either being sufficient for the repression of PAX5 transactivity by PAX5‐C. We performed ChIP‐qPCR under conditions of the luciferase assay and inferred that these two mechanisms involved the inhibition of direct binding of PAX5 to the promoter due to promoter occupancy by PAX5‐C, and recruitment of HDAC1/3 to the PAX5 transcription complex by the binding of PAX5‐C to PAX5 on the promoter. The present results provide novel insight into the mechanisms of how PAX5‐fusion proteins inhibit PAX5 function.

## Graphical abstract

PAX5::CBFA2T3 (PAX5‐C) is a fusion protein of the B‐cell transcription factor, PAX5, and is found in B‐cell ALL. We propose a putative model of two‐way inhibition of PAX5 transcriptional activity by PAX5‐C. There are two ways of repression by PAX5‐C: DNA‐binding‐dependent way and HDAC‐dependent way, with either being sufficient for the repression. HDAC recruitment to PAX5‐C is not required for the DNA‐binding‐dependent way.

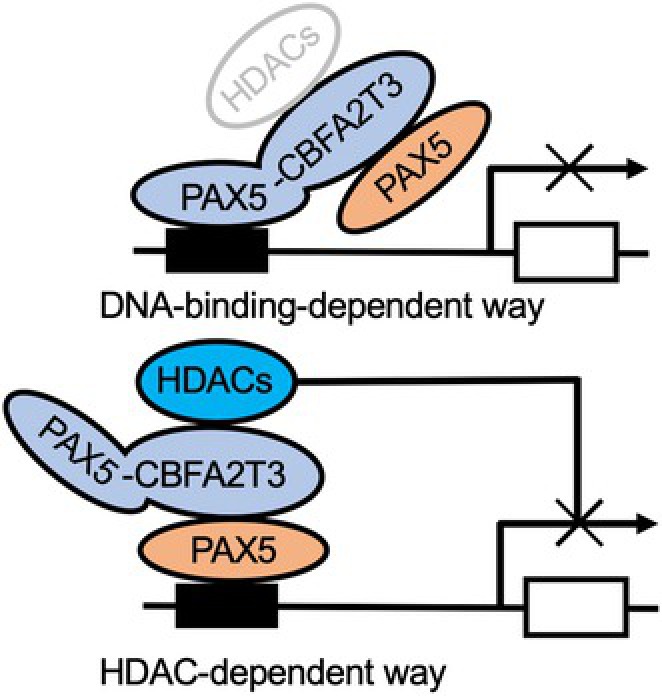

**Abbreviations**
AML: acute myeloid leukemia
B‐ALL: B‐cell precursor acute lymphoblastic leukemia
BLNK: B‐cell linker protein
CBFA2T: core‐binding factor alpha subunit 2 translocated to
ChIP: chromatin immunoprecipitation
DOX: doxycycline
ETO: eight twenty‐one
GCN5: general control of amino acid synthesis 5
HAT: histone acetylase
HDAC: histone deacetylase
IB: immunoblot
IP: immunoprecipitation
NcoR: nuclear receptor corepressor
NHR: nervy homology region
PCR: polymerase chain reaction
PAX5: paired box gene 5
PAX5‐C: PAX5::CBFA2T3
P/CAF: p300/CBP‐associated factor
PD: paired domain
qPCR: quantitative PCR
RUNX1: runt‐related transcription factor 1
RUNX1T1: runt‐related transcription factor 1 translocated to 1
RUNX1‐R: RUNX1::RUNX1T1
SEM: standard error
SIN3A: SIN3 transcription regulator family member 3A
SMRT: silencing mediator for retinoid and thyroid hormone receptors
TIP60: TAT‐interacting protein 60‐kD
TSA: trichostatin A

PAX5 is a member of the PAX family of transcription factors. In the hematopoietic system, PAX5 is expressed exclusively in the B‐lymphoid lineage until the onset of plasma cell differentiation and is essential for not only promoting B‐cell differentiation but also maintaining B‐cell identity [1]. PAX5 promotes B‐cell differentiation by transcriptional activation of B‐lineage‐specific genes, such as CD19, CD79A, and BLNK [2, 3, 4, 5].

Gene alterations in PAX5, including deletions, point mutations, and amplifications, occur in approximately 30% of B‐ALL cases, and chromosomal rearrangements account for 2–3% [6, 7, 8]. Most rearrangements lead to the expression of in‐frame fusion transcripts. They encode chimeric proteins composed of the N‐terminal portion of PAX5 and the C‐terminal region of a variety of heterogeneous fusion partners, including transcription factors, kinases, and structural proteins [8, 9, 10, 11]. With the exception of PAX5::JAK2, almost all PAX5 fusion proteins examined to date had a dominant‐negative effect on the transcriptional activity of PAX5 [7, 12, 13, 14, 15, 16, 17]. Furthermore, the expression of *PAX5* fusion genes, such as *PAX5::PML, PAX5::ETV6, PAX5::FOXP1*, and *PAX5::ENL*, in mouse hematopoietic cells caused B‐ALL [18, 19, 20]. These findings indicate that *PAX5* rearrangements play an important role in B‐ALL development through the impairment of PAX5 function; however, detailed causative mechanisms of the dominant‐negative effects of PAX5 fusion proteins on PAX5 transcriptional activity have yet to be clarified.

CBFA2T3, also known as ETO2, belongs to the ETO family together with RUNX1T1, also known as CBFA2T1 or ETO. ETO family members are transcriptional repressors that interact with transcription factors bound to promoters of target genes and various repressor proteins, including NCoR, SMRT, SIN3a, and HDACs [21, 22, 23]. Each of these proteins has four NHR domains and forms homo‐ or hetero‐oligomeric ETO complexes via the NHR2 domain [24, 25].

Translocations of ETO family members with RUNX1 are recurrently found in patients with AML. Particularly, RUNX1::RUNX1T1 (RUNX1‐R) is one of the most frequent fusion genes in AML. RUNX1 is a key transcription factor for definitive hematopoiesis and regulates a wide range of genes essential for hematopoiesis, including growth factor receptors, enzymes, and cytokines [26, 27]. RUNX1‐R may have a critical and initiating role in the processes that can lead to leukemic transformation, possibly through suppressing normal RUNX1 function in a dominant‐negative manner to cause myelodysplasia or through inhibiting apoptosis of hematopoietic progenitors [28, 29, 30]. RUNX1‐R and RUNX1::CBFA2T3 selectively bind to HDAC1 and HDAC3 among HDACs [23, 31]. The recruitment of HDACs into the transcriptional complex through RUNX1 fusion partners is the expected mechanism behind the dominant‐negative effect on the transcriptional activity of RUNX1.

The fusion gene between *PAX5* and *CBFA2T3*, *PAX5::CBFA2T3* (*PAX5‐C*), is found in about 0.3–0.8% of B‐ALL cases [9, 11]. The function of PAX5‐C has not been analyzed at all, including whether it has a dominant‐negative effect on PAX5 transcriptional activity; however, it is predicted that PAX5‐C, like other PAX5 fusion proteins, represses PAX5 transcriptional activity and that PAX5‐C, like RUNX1::CBFA2T3, represses PAX5 transcriptional activity by recruiting HDACs to the PAX5 transcriptional complex through the CBFA2T3 region. To test this, we examined the presence of the repression of PAX5 transcriptional activity by PAX5‐C, the requirement for HDAC for the repression, and mechanisms of repression other than HDAC recruitment.

## Materials and methods

### Plasmids

Flag‐PAX5/pcDNA, PAX5/pcDNA, Flag‐PAX5 M/pcDNA, and BLNK‐luc/pGL4 were described previously [15, 18]. Myc‐His‐HDAC1/pcDNA, HDAC3/pcDNA, HDAC4/pcDNA, HDAC5/pcDNA, and HDAC6/pcDNA were also previously reported [32]. Halo‐KAT2A/pFN21A, Halo‐KAT6A/pFN21A, and Halo‐KAT5/pFN21A were obtained from Promega (Madison, WI, USA). Flag‐GCN5/pEBP and Flag‐P/CAF/pCI were from Addgene (Cambridge, MA, USA). 3xFlag‐TIP60/pcDNA was made by insertion of the PCR‐amplified TIP60 coding region into 3xFlag/pcDNA using In‐Fusion^®^HD Cloning Kit (Takara Bio Inc., Kusatsu, Japan).

The coding region of PAX5‐C was amplified by PCR from a patient's cDNA, cloned into N‐Flag/pcDNA [33] at EcoRV and NotI sites using In‐Fusion^®^HD Cloning Kit for Flag‐PAX5‐C/pcDNA. PAX5‐C/pcDNA was prepared by deletion of the Flag‐coding region from Flag‐PAX5‐C/pcDNA by digestion with KpnI and EcoRI‐HF and blunt‐end ligation. Flag‐PAX5 M‐C/pcDNA was made by swapping the PD of PAX5 from Flag‐PAX5 M/pcDNA to Flag‐PAX5‐C/pcDNA using In‐Fusion^®^HD Cloning Kit. Flag‐PAX5 ΔC/pcDNA and Flag‐ΔN CBFA2T3/pcDNA were made by deletion mutagenesis using KOD‐Plus‐Mutagenesis Kit (TOYOBO, Osaka, Japan).

All vectors produced were used in experiments after Sanger sequencing of the entire coding region to confirm the absence of mutations due to PCR errors. Primers used for expression vector construction are listed in Table S1.

### Antibodies

Anti‐PAX5 N‐ter antibody (sc‐1975) and anti‐PAX5 C‐ter antibody (sc‐1974) can detect the N‐terminal region of PAX5 commonly contained in PAX5 and PAX5‐C and the C‐terminal region contained in PAX5 only, respectively, and they were purchased from Santa Cruz Biotechnology (Dallas, TX, USA). Anti‐Flag antibody (M2) was from Sigma Aldrich (St. Louis, MO, USA). Anti‐β‐actin antibody was obtained from Bio Vision (Milpitas, CA, USA). Anti‐HDAC1 antibody (34589S), anti‐HDAC3 antibody (85057S), anti‐HDAC4antibody (2072S), anti‐HDAC5 antibody (2082S), and anti‐HDAC6 antibody (2162S) were purchased from Cell Signaling Technology (Danvers, MA, USA). Anti‐HaloTag^®^ monoclonal antibody was from Promega. Anti‐CBFA2T3 antibody (17190‐1‐AP) was obtained from Proteintech (Rosemont, IL, USA).

### Cell culture

Human embryonic kidney (HEK) 293 T was maintained in Dulbecco's Modified Eagle Medium with high‐level glucose (Wako, Osaka, Japan) containing 10% fetal bovine serum (FBS). NALM6, a *DUX4‐IGH*‐positive B‐ALL cell line, was maintained in Roswell Park Memorial Institute (RPMI) 1640 (Wako) containing 10% FBS.

### Luciferase assay

The luciferase assay was performed using the Dual‐Luciferase Reporter Assay System (Promega, Fitchburg, WI, USA), according to the manufacturer's instructions. Briefly, HEK 293 T (1.0 × 10^4^) were transfected with the reporter gene (112.5 ng), Renilla luciferase expression vector phRG–TK (12.5 ng) for reference, and expression vectors of the protein of interest (125 ng each). Luciferase luminescence was measured with the GloMax^®^ Navigator Microplate Luminometer (Promega). Experiments were carried out at least three times in duplicate.

### Immunoblot

HEK 293 T were lysed with sample buffer (125 mm Tris–HCl pH 6.8, 2% SDS, 10% glycerol, 2.5% 2‐mercaptoethanol and 0.04% bromophenol blue) and subjected to SDS/PAGE using 8–10%‐SDS/polyacrylamide gels followed by immunoblot. Primary and secondary antibodies were used at ×1000 and ×2000 dilution, respectively. Signals were visualized with ECL Prime Western Blotting Detection Reagent (Cytiva, Tokyo, Japan) and Light Capture II (Atto Corporation, Tokyo, Japan).

### Transient transfection

Transient transfection of HEK293T in the luciferase assay was performed with FuGENE^®^ (Promega, Madison, WI, USA) according to the manufacturer's instructions. Transient transfections in other experiments were performed by lipofection using PEI Max (MW40,000) (Polysciences, Warrington, PA, USA) based on a previous report [34].

### Knockdown

Expression vectors of shRNA were prepared by inserting a DNA fragment for shRNA expression designed according to the manual of Riken BRC (Tsukuba, Japan) into the Dox‐inducible shRNA expression vector CS‐RfA‐ETR. This vector was provided by RIKEN BRC through the National BioResource Project of MEXT, Japan (cat. RDB08020). shRNA target sequences were selected from previous reports [35] or target sequences of MISSION™ shRNA (Merck & Co., Inc., Rahway, NJ, USA) and are summarized in Table S2. All vectors were confirmed to show insert incorporation and to be free of mutations due to PCR errors by Sanger sequencing. Dox (1 μg·mL^−1^) was added 24 h after transfection, and cells were lysed 48 h later.

### ChIP‐qPCR

ChIP‐qPCR was performed using the SimpleChIP Enzymatic Chromatin IP Kit (Cell Signaling Technology, Danvers, MA, USA) according to the manufacturer's instructions. As in the luciferase assay, 3.0 × 10^6^ 293 T cells were transfected with BLNK‐luc/pGL4, various expression vectors, and an empty vector, and used for the experiments 48 h later. Normal mouse IgG (sc‐2025, Santa Cruz, Dallas, TX, USA) and anti‐Flag antibody were used for precipitation, respectively. We used the cyber green method to quantify the immunoprecipitated DNA fragment. The primers used in qPCR are shown in Table S1 (BLNK‐luc PCR). Experiments were performed at least three times in duplicate.

### Statistical analysis

Student's *t*‐test was used for comparisons of two experimental data groups with equal variances. Welch's *t*‐test was applied for those without equal variances. *, **, and ns indicate *P* < 0.05, *P* < 0.01, and not significant, respectively.

## Results

### PAX5‐C dominant negatively repressed the transcriptional activity of PAX5

**Fig. 1:**
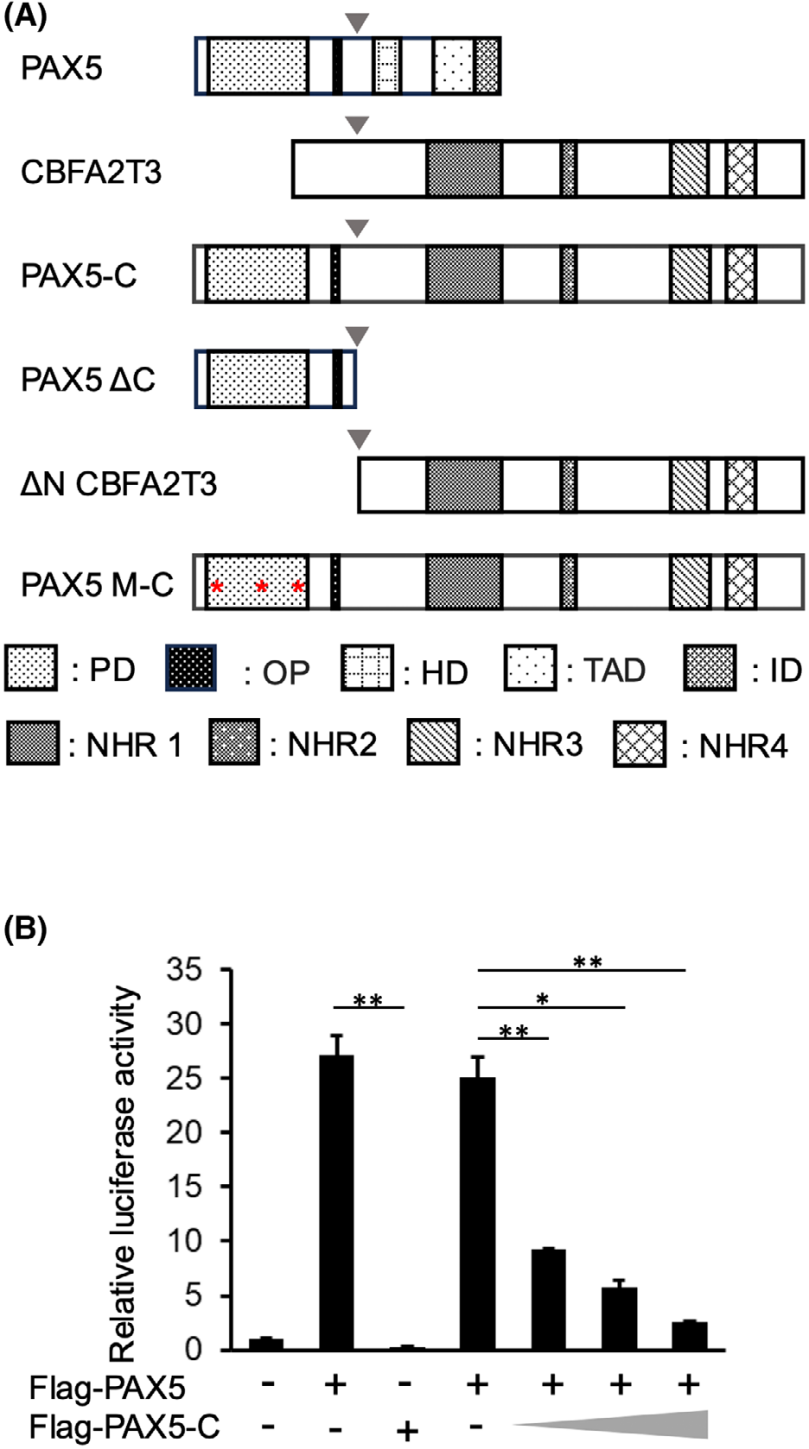
Dominant‐negative effect of PAX5–C on the transcriptional activity of PAX5. (A) Structures of PAX5, CBFA2T3, PAX5‐C, and their mutants. PAX5 ΔC and ΔN CBFA2T3 are PAX5 and CBFA2T3 portions of PAX5‐C, respectively. The breakpoint is indicated by arrowheads. Red asterisks indicate the point mutations. PD, paired box domain; OP, octapeptide; HD, partial homeodomain; TAD, transactivation domain; ID, inhibitory domain; NHR, nervy homology region. (B) Dominant‐negative transcriptional repression by PAX5–C. Luciferase assays were performed by transfection with the indicated expression vectors and BLNK‐luc/pGL4, a reporter gene, into HEK293T cells. PAX5‐C expression vectors were used with a three‐step increase at 25%, 50%, and 100% of PAX5. Luciferase activities are shown as values relative to the basal activity in cells transfected with only the reporter gene. Experiments were performed biologically independently three times in duplicate, and mean values are plotted on bar charts. Error bars indicate SEM. Student's *t*‐test was used to test the significance of difference. *P < 0.05; **P < 0.01.

We created the expression vector of PAX5‐C, in which PAX5 exon 5 was fused with CBFA2T3 exon 2. The protein structure of this fusion protein is shown in Fig. 1A. In PAX5‐C, the PD of PAX5, which is important for DNA binding, is retained, but its C‐terminal region required for transcriptional activation or repression is lost. CBFA2T3 retains almost all regions. The luciferase assay showed that PAX5‐C exhibited no transcriptional activity and dominant negatively repressed the transcriptional activity of PAX5 (Fig. 1B). Protein expression from Flag‐PAX5‐C/pcDNA was confirmed by immunoblot (Fig. S1).

### PAX5‐C is associated with HDAC1 and HDAC3, but neither knockdown nor inhibition of them was sufficient to release the repression of PAX5 transactivity by PAX5‐C

**Fig. 2:**
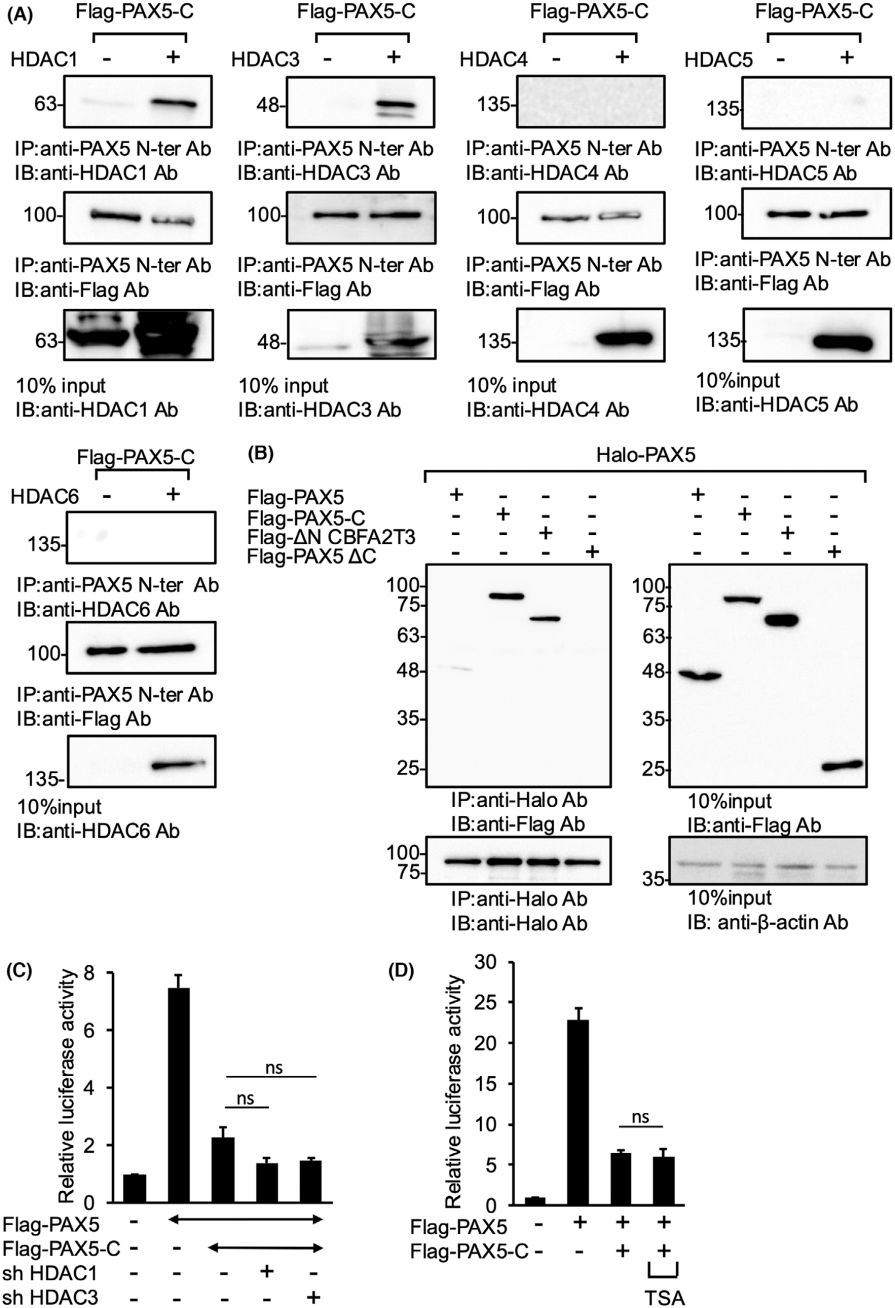
PAX5‐C associated with HDAC1/3, but neither knockdown nor inhibition of them was sufficient to release the repression of PAX5 transactivity by PAX5‐C. (A) Interaction between PAX5‐C and HDAC1/3. The indicated expression vectors were used to transfect HEK293T. Cell lysates were subjected to IP and IB with the indicated antibodies. The numbers on the left indicate the position of molecular‐weight (kDa) markers. Anti‐PAX5 N‐ter Ab recognizes both PAX5 and PAX5‐C. Experiments were performed biologically independently two times. (B) PAX5 interacted with PAX5‐C but not PAX5 itself. Co‐IP was performed as in (A). Experiments were performed biologically independently two times. (C) Knockdown of HDAC1/3 did not attenuate the repression of PAX5 transactivity by PAX5‐C. Luciferase assays were performed as in Fig. 1B with cotransfection of the indicated shRNA expression vectors. The plasmid for PAX5‐C was used at 25% of the amount of PAX5. All cells were treated with DOX (1 μg·mL^−1^) 24 h prior to cell lysis. Experiments were performed biologically independently three times in duplicate, and mean values are plotted on bar charts. Error bars indicate SEM. Student's *t*‐test was used to test the significance of difference. ns: not significant. (D) The class I HDAC inhibitor TSA did not affect the repression of PAX5 transactivity by PAX5‐C. The luciferase assay was performed as in Fig. 1B. TSA (100 nm) was added to the indicated samples 24 h prior to cell lysis. Experiments were performed biologically independently three times in duplicate, and mean values are plotted on bar charts. Error bars indicate SEM. Student's *t*‐test was used to test the significance of difference. ns: not significant.

We examined the association of PAX5‐C with HDACs by co‐IP using anti‐PAX5 N‐ter antibody for IP; similar to reports for RUNX1::CBFA2T3, another fusion protein of CBFA2T3 [31], HDAC1 and HDAC3 (HDAC1/3) bound to PAX5‐C but HDAC4, HDAC5, and HDAC6 did not (Fig. 2A). IP by control IgG showed no nonspecific band that could be confused with HDAC1 or HDAC3 (Fig. S2A). For further confirmation, we conducted co‐IP using different antibodies for IP and observed similar results (Fig. S2B). Furthermore, PAX5 showed no association with PAX5 itself but a strong interaction with PAX5‐C, suggesting that PAX5‐C bound to PAX5 through its CBFA2T3 portion. Consistent with this result, its CBFA2T3 portion, ΔN CBFA2T3, rather than its PAX5 portion, PAX5 ΔC, associated with PAX5 (Fig. 2B). These results indicate that HDAC1/3 were recruited into the PAX5 transcription complex through mediation by PAX5‐C.

To investigate the requirement for HDAC1/3 in the repression of PAX5 transactivity by PAX5‐C, we performed HDAC knockdown. We generated 2 and 4 shRNA expression vectors for HDAC1 and HDAC3, respectively, and examined their knockdown effects (Fig. S3A). The most effective vectors for each HDAC were used in subsequent experiments. Luciferase assays revealed that knockdown of any HDAC had no effect on the repression of PAX5 transactivity by PAX5‐C (Fig. 2C), although the knockdown effect might be insufficient, especially in HDAC3 knockdown (Fig. S3B). Since HDAC knockdown was not sufficient, we also conducted experiments using TSA, an inhibitor of Class 1 HDACs. Also, TSA did not significantly attenuate the repression of PAX5 transactivity by PAX5‐C (Fig. 2D). These data suggest that neither knockdown nor inhibition of HDAC1/3 was sufficient to release the repression of PAX5 transactivity by PAX5‐C.

**Fig. 3:**
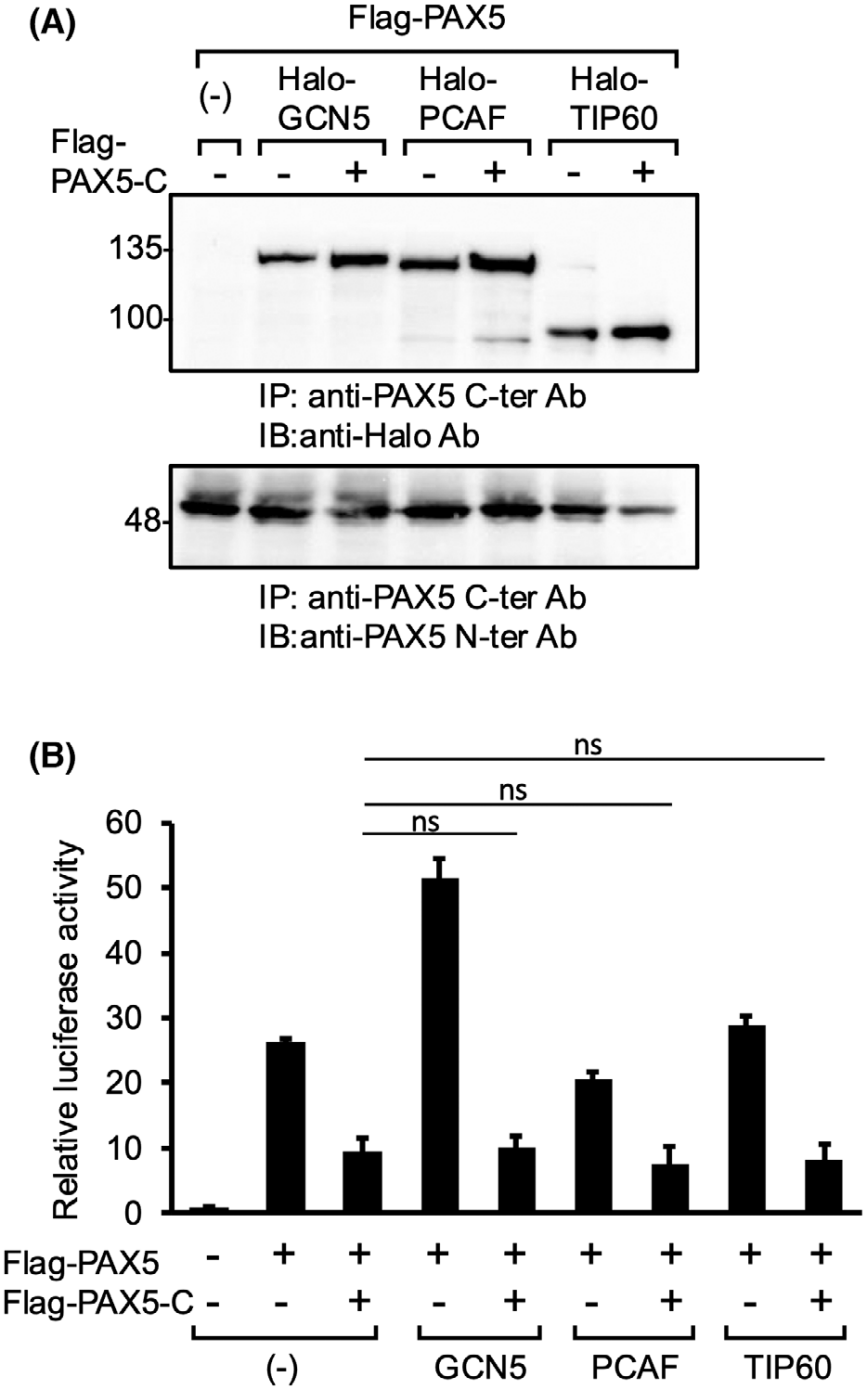
Co‐expression of PAX5‐C did not affect the interaction of PAX5 with HATs. (A) Interaction between PAX5 and HATs. Co‐IP was performed as in Fig. 2A. (B) Co‐expression of HATs did not attenuate the repression of PAX5 transactivity by PAX5‐C. The luciferase assay was performed as in Fig. 1B. Experiments were performed biologically independently three times in duplicate, and mean values are plotted on bar charts. Error bars indicate SEM. Student's *t*‐test was used to test the significance of difference. ns: not significant.

It was reported that the transcriptional activity of PAX5 was enhanced by its association with HATs such as GCN5 [36]. This raised the possibility that PAX5‐C suppresses the transcriptional activity of PAX5 by inhibiting the association between PAX5 and HATs. This was examined by co‐IP. PAX5 bound to GCN5, P/CAF, and TIP60, but such binding was not attenuated by PAX5‐C co‐expression (Fig. 3A). Furthermore, co‐expression of any HAT did not significantly attenuate the repression of PAX5 transactivity by PAX5‐C in the luciferase assay (Fig. 3B).

### Both DNA‐binding and HDAC activity needed to be inhibited to release the repression of PAX5 transactivity by PAX5‐C

**Fig. 4:**
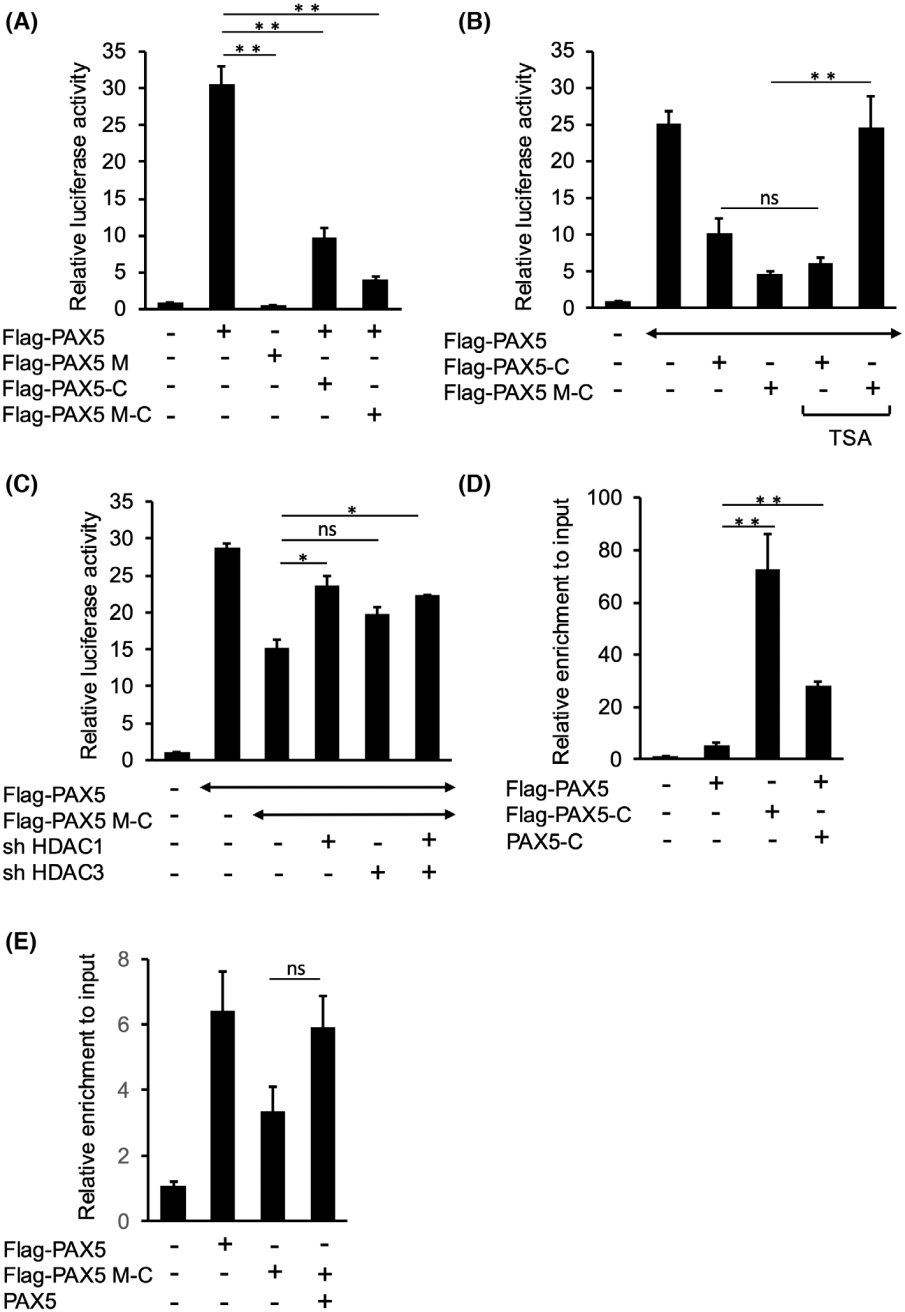
Both DNA‐binding and HDAC activity needed to be inhibited to release the repression of PAX5 transactivity by PAX5‐C. (A) Loss of DNA‐binding ability did not affect the repression of PAX5 transactivity by PAX5‐C. Luciferase assays were performed as in Fig. 1B. Experiments were performed biologically independently three times in duplicate, and mean values are plotted on bar charts. Error bars indicate SEM. Student's *t*‐test was used to test the significance of difference. **P < 0.01. (B) PAX5 inhibition by PAX5 M‐C was released by the HDAC inhibitor. The luciferase assay was performed as in Fig. 2D. Experiments were performed biologically independently three times in duplicate, and mean values are plotted on bar charts. Error bars indicate SEM. Student's *t*‐test was used to test the significance of difference. **P < 0.01; ns: not significant. (C) PAX5 inhibition by PAX5 M‐C was attenuated by knockdown of HDAC1 or HDAC3. The luciferase assay was performed as in Fig. 2D. Experiments were performed biologically independently three times in duplicate, and mean values are plotted on bar charts. Error bars indicate SEM. Student's *t*‐test was used to test the significance of difference. *P < 0.05; ns: not significant. (D) DNA‐binding ability of PAX5‐C was much stronger than that of PAX5, and promoter‐binding of PAX5 was significantly increased by PAX5‐C co‐expression. BLNK‐luc/pGL4 and the indicated expression vectors were used to transfect HEK293T. ChIP‐qPCR using anti‐Flag antibody was performed to detect the binding of Flag‐PAX5 or Flag‐PAX5‐C to the promoter region of BLNK‐luc/pGL4. Enrichments of the target DNA fragments by anti‐Flag antibody were normalized to the input and plotted on bar charts. Experiments were performed biologically independently three times in duplicate, and mean values are plotted on bar charts. Error bars indicate SEM. Student's *t*‐test was used to test the significance of difference. **P < 0.01. (E) Promoter‐binding of PAX5 M‐C was considered to be increased by PAX5 co‐expression. ChIP‐qPCR was performed as in (A). Experiments were performed biologically independently three times in duplicate, and mean values are plotted on bar charts. Error bars indicate SEM. Student's *t*‐test was used to test the significance of difference. ns: not significant.

We examined the importance of the DNA‐binding ability of PAX5‐C in the suppression of PAX5 transcriptional activity using PAX5 M and PAX5 M‐C, which had three DNA‐binding‐defective mutations: P80R, V26G, and NDTVP126_130RA [9], within PD of PAX5 and PAX5‐C, respectively (Figs 1A, S4). In the luciferase assay, the transcriptional activity of PAX5 M was markedly reduced, likely due to impaired DNA‐binding ability (Fig. 4A). Unexpectedly, PAX5 M‐C showed a similar level of repression of PAX5 transactivity as PAX5‐C (Fig. 4A). These results indicate that inhibition of PAX5‐C DNA binding was insufficient to release PAX5‐C‐mediated suppression of PAX5 transcriptional activity.

Then, we investigated whether the repression of PAX5 transcriptional activity by PAX5 M‐C required HDAC activity using TSA and shRNA against HDAC1/3. It was almost completely abolished by TSA (Fig. 4B) and attenuated by knockdown of HDAC1 (Fig. 4C). The change induced by knockdown of HDAC3 was only marginally significant (*P* = 0.05154). These findings suggest that the transcriptional repression by PAX5 M‐C was dependent on the recruitment of HDAC1/3 through PAX5 M‐C. Given that transcriptional repression by PAX5‐C with intact DNA‐binding ability was not attenuated at all by TSA or knockdown of HDAC1/3, PAX5‐C will also have a HDAC‐independent and DNA‐binding‐dependent mechanism for the repression of PAX5 transcriptional activity in addition to a HDAC‐dependent mechanism. These findings indicate that there are two mechanisms facilitating the repression of PAX5 transactivity by PAX5‐C: DNA‐binding‐dependent and HDAC‐dependent mechanisms, with either being sufficient for repression of PAX5 transactivity by PAX5‐C; therefore, both DNA‐binding and HDAC activity need to be inhibited to release PAX5‐C‐mediated suppression of PAX5 transcriptional activity.

To clarify the mechanism of DNA‐binding‐dependent repression of PAX5 transactivity by PAX5‐C in the luciferase assay, the reporter gene‐binding ability of PAX5‐C was examined by ChIP‐qPCR. PAX5‐C showed stronger binding to the reporter gene than PAX5, suggesting that PAX5‐C repressed PAX5 transcriptional activity by competitive inhibition of promoter binding of PAX5 (Fig. 4D); however, co‐expression of PAX5‐C significantly increased promoter binding of PAX5. This result was unexpected; however, taking the association between PAX5 and PAX5‐C (Fig. 2B) into account, it suggests that in the presence of PAX5‐C, PAX5 indirectly binds to the promoter through association with PAX5‐C bound to the promoter in addition to direct binding to it. In addition, since PAX5 could not exert transcriptional activity regardless of HDAC inhibition under this condition (Fig. 2C,D), this indirect promoter binding will be insufficient for transcriptional activation. That is, the DNA‐binding‐dependent mechanism of PAX5 repression by PAX5‐C will be inhibition of direct promoter binding of PAX5 facilitated by promoter occupation by PAX5‐C.

This model, indirect promoter binding of PAX5 through association with PAX5‐C bound to the promoter, provided a clue to answering the question of how PAX5 M‐C represses PAX5 transactivity despite its loss of DNA‐binding ability. That is, PAX5 M‐C might indirectly bind to a promoter through association with PAX5 bound to the promoter. To test this hypothesis, the promoter‐binding ability of PAX5 M‐C was examined by ChIP‐qPCR. The promoter binding of PAX5 M‐C was markedly reduced to the level under promoter binding of PAX5. However, co‐transfection of PAX5 was considered to increase it (Fig. 4E). Although the difference was not significant, this result supported the above hypothesis.

**Fig. 5:**
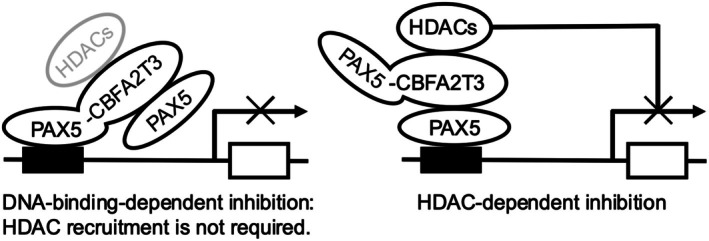
Schematic presentation of the putative model of two‐way inhibition of PAX5 transcriptional activity by PAX5‐C.

In summary, PAX5‐C may have two mechanisms for repression of PAX5 transactivity: One is promoter occupation by PAX5‐C and the other is HDAC‐recruitment to a transcriptional complex through association of PAX5‐C with PAX5 bound to the promoter. Schemas for these putative models are presented in Fig. 5.

## Discussion

In the present study, we show that there are two mechanisms by which PAX5‐C dominant‐negatively represses PAX5 transcriptional activity: One is DNA‐binding‐dependent and the other is HDAC‐dependent. Regarding the former, it may be important for the DNA‐binding ability of PAX5‐C to be stronger than that of PAX5, and for the latter, it may be necessary for PAX5‐C to bind to HDAC1/3 (Fig. 5). Since a common point among the diverse PAX5 fusion proteins is for the PAX5 portion to contain PD for DNA‐binding, it has been predicted that a common mechanism to explain the repression of PAX5 transactivity is the occupation of the PAX5 target promoter by PAX5 fusion proteins. However, although PAX5::C20S and PAX5::ETV6 have been reported to show stronger DNA‐binding ability than PAX5 [13], another study examining the DNA‐binding ability of various PAX5 fusion proteins by EMSA and ChIP‐qPCR revealed that many of them had weaker DNA‐binding ability than PAX5 [16], raising the possibility that promoter occupation was not the only mechanism. Since the PAX5‐binding ability of PAX5 fusion protein was suggested to depend on its C‐terminal fusion partner (Fig. 2B) and this ability has not been examined in most PAX5 fusions to date, it is unclear whether PAX5‐binding is a common feature of PAX5 fusion proteins. However, there is a possibility that it is unexpectedly common. If so, PAX5 fusion proteins with PAX5‐binding ability can interact with promoter‐bound PAX5 even if they have weak DNA‐binding ability and can repress its transcriptional activity by various mechanisms characteristic of C‐terminal fusion partners.

Our findings may provide clues for the development of therapeutic agents targeting another fusion protein of the ETO family, RUNX1‐R. HDACs have been considered to be good therapeutic targets for releasing the repression of RUNX1 transcriptional activity by RUNX1‐R (see Introduction) [37]. Many attempts were made to induce differentiation of RUNX1‐R‐positive AML with HDAC inhibitors, but they have not been applied for clinical use to date. Similar to PAX5‐C, RUNX1‐R might also have a mechanism for inhibiting RUNX1 DNA‐binding in addition to the HDAC‐dependent mechanism.

In the present study, as a first step to elucidate the mechanism of inhibition of PAX5 function by PAX5‐C, we used only a simple model, a luciferase assay model in 293 T, where PAX5‐C inhibited simplified PAX5‐dependent transcription; however, the luciferase assay cannot accurately reproduce transcriptional regulation by transcription factors. Therefore, the mechanism proposed by the luciferase assay needs to be verified under more physiological conditions. In future studies, it will be necessary to verify whether the proposed model is also valid in B lymphocytes, in which endogenous PAX5 and its cofactors co‐operatively regulate transcription from DNA wrapped around chromatin. In particular, it is important to examine the ability of PAX5‐C and PAX5 M‐C to cause ALL *in vivo* and compare the efficacy of HDAC inhibitors for the treatment of ALL developed by them.

## Conflict of interest

The authors declare no conflict of interest.

## Peer review

The peer review history for this article is available at https://www.webofscience.com/api/gateway/wos/peer‐review/10.1002/2211‐5463.70087.

## Author contributions

FH conceptualized the study. FH, RU, and TY conducted data curation and formal analysis. FH, RU, AT, YI, YK, YK, MI, KO, MN, OS, TS, and KH contributed to the investigation and methodology in this study. FH and RU wrote the original draft, and all others reviewed and edited it. All authors approved the final manuscript for submission.

## Supporting information


**Table S1.** List of PCR primers.
**Table S2.** List of target sequences of shRNA.
**Fig. S1.** Expression of PAX5 and PAX5‐C from expression vectors.
**Fig. S2.** Control experiments for co‐IP between PAX5‐C and HDACs.
**Fig. S3.** Knockdown effect of HDAC shRNA.
**Fig. S4.** Expression of PAX5, PAX5‐C, and PAX5 M‐C from expression vector.

## Data Availability

The data that support the findings of this study are available from the corresponding author upon reasonable request.
