# Supplementary material for: Two‐way inhibition of PAX5 transcriptional activity by PAX5::CBFA2T3
Source: FEBS Open Bio. 2025 Jul 11;15(11):1789–99. doi: 10.1002/2211-5463.70087 (PMC12582980; doi:10.1002/2211-5463.70087)
Supplement: Supplementary file 1 — Table S1. List of PCR primers. Table S2. List of target sequences of shRNA. Fig. S1. Expression of PAX5 and PAX5‐C from expression vectors. Fig. S2. Control experiments for co‐IP between PAX5‐C and HDACs. Fig. S3. Knockdown effect of HDAC shRNA. Fig. S4. Expression of PAX5, PAX5‐C, and PAX5 M‐C from expression vector. [file FEB4-15-1789-s001.docx]

**Supporting Information**

**Fig. S1** Expression of PAX5 and PAX5-C from expression vectors. The indicated expression vectors were used to transfect HEK293T. PAX5-C expression vectors were used with a three-step increase at 25, 50, and 100% of PAX5. Cell lysates were subjected to IB with the indicated antibodies. The numbers on the left indicate the position of molecular-weight (kDa) markers. Black and gray arrows indicate PAX5-C and PAX5, respectively.

**Fig. S2** Control experiments for co-IP between PAX5-C and HDACs. (A) Control IP by control IgG. The indicated expression vectors were used to transfect HEK293T. Cell lysates were subjected to IP and IB with the indicated antibodies as in Fig. 2A. The numbers on the left indicate the position of molecular-weight (kDa) markers. The band indicated by asterisk is non-specific band. (B) Alternative IP to confirm the result of Fig. 2A. IP and IB was conducted as in Fig. 2A using different antibodies from Fig. 2A. The bands indicated by asterisks are non-specific bands.

**Fig. S3** Knockdown effect of HDAC shRNA. (A) Selection of shRNAs. The expression vector of the indicated HDACs and DOX-inducible shRNA expression vectors were used to transfect HEK293T. DOX (1 μg/mL) was added 24 hours before cell lysis, and IB was performed as in Figure 1B. The shRNAs with the strongest knockdown effect are shown in bold and were used for subsequent experiments. (B) Knockdown effect of selected shRNA in the condition of luciferase assay in Fig. 2C. Transfection and IB were conducted as in (A).

**Fig. S4** Expression of PAX5, PAX5-C, and PAX5 M-C from expression vector. IB was performed as in Fig. S1.
